# Supplementary material for: Common dysregulation network in the human prefrontal cortex underlies two neurodegenerative diseases
Source: Mol Syst Biol. 2014 Jul 30;10(7):743. doi: 10.15252/msb.20145304 (PMC4299500; doi:10.15252/msb.20145304)
Supplement: Supplementary file 1 — Supplementary Information [file msb0010-0743-sd1.pdf]

Supplementary Information for

Common Dysregulation Network in the Human Prefrontal Cortex underlies Two Neurodegenerative Diseases

Manikandan Narayanan[1,*], Jimmy L. Huynh[2,3], Kai Wang[4], Xia Yang[5], Seungyeul Yoo[3], Joshua McElwee[4], Bin Zhang[3], Chunsheng Zhang[4], John R. Lamb[4], Tao Xie[4], Christine Suver[6], Cliona Molony[4], Stacey Melquist[4], Andrew D. Johnson[7], Guoping Fan[8], David J. Stone[4], Eric E. Schadt[3], Patrizia Casaccia[2,3], Valur Emilsson[9,10], Jun Zhu[3,*]

[1]National Institute of Allergy and Infectious Diseases, Bethesda, MD, USA; [2]Department of Neuroscience, Icahn School of Medicine at Mount Sinai, NY, USA; [3]Department of Genetics and Genomic Sciences, Icahn School of Medicine at Mount Sinai, NY, USA; [4]Merck Research Laboratories, Merck & Co., Inc., USA; [5]Department of Integrative Biology and Physiology, University of California at Los Angeles, CA, USA; [6]Sage Bionetworks, Seattle, WA, USA; [7]National Heart, Lung and Blood Institute, Bethesda, MD, USA; [8]Department of Human Genetics, University of California at Los Angeles, CA, USA; [9]Icelandic Heart Association, Kopavogur, Iceland; [10]Faculty of Pharmaceutical Sciences, University of Iceland, Reykjavik, Iceland

*Address correspondence to:

Dr. Manikandan Narayanan
Laboratory of Systems Biology
National Institute of Allergy and Infectious Diseases
Bethesda, MD 20892
manikandan.narayanan@nih.gov

Dr. Jun Zhu
Department of Genetics and Genomic Sciences
Institute for Genomics and Multiscale Biology
Mount Sinai School of Medicine
New York, NY 10029
jun.zhu@mssm.edu

A. Supplementary Text

A.1 DC analysis complements differential expression (DE) analysis

Many microarray studies focus on differential expression (DE), while few have explored the differential co-expression (DC) of gene expression traits. We observed in the main text that our DC analysis detected disease-associated genes that were not found by conventional t-statistics based DE analysis in both AD and HD comparisons to controls. Here we additionally examined if the differential expression (DE) P-values of the reporters detected as DC pairs had any bias towards being GOC or LOC pairs, and found that reporters involved in GOC pairs compared to LOC pairs are more likely to be DE. In detail, as **Suppl Figures S1A and S2** show, more GOC pairs compared to LOC pairs are associated with reporter pairs whose DE is high in the AD vs. controls comparison. For instance, using a lenient (uncorrected) DE P-value cutoff of 0.05, we found that 9.20% of all 18,560 GOC pairs were between two DE reporters, whereas only 0.46% of all 9,663 LOC pairs were between two DE reporters (these values are 37.33% and 17.25% respectively if we require only one reporter in the GOC or LOC pair to be DE). We observed a similar trend for the HD vs. controls comparison (**Suppl Figures S1B and S3**).

A.2 Dysregulation among known genetic causes of AD and HD

Several common variants have been convincingly shown to associate with AD (www.genome.gov catalog accessed in May 2011 and listed in **Suppl Table S2**). In addition to common variants, rare variants in *PSEN1*, *PSEN2*, and *APP* have been identified through Mendelian inheritance (OMIM at http://omim.org/). Variants across the *APOE* gene are the best supported genetic causes of AD and those with the greatest effect sizes (Lopez et al, 1998; Tsai et al, 1994), while weaker effects across a number of other loci have been reported (Beecham et

al, 2009; Bertram et al, 2008; Carrasquillo et al, 2009; Harold et al, 2009; Heinzen et al, 2009; Kramer et al, 2010; Reiman et al, 2007). The allelic effects for *APOE*, *GAB2*, *SASH1* and *FAM113B* related genotypes on AD risk were replicated in the HBTRC (Harvard Brain Tissue Resource Center) samples (**Suppl Table S2**). The most likely reason for lack of replication of other reported risk variants (**Suppl Table S2**) is lack of power in our relatively small study group. We find that nine of these AD-related genes show at least one pair of DC in AD, with *SASH1*, *APOE* and *ABCA7* participating in the greatest number of DC gene pairs (**Table 2** in the main text). Strikingly, the majority (69%) of the DC gene pairs involving the AD causing genes showed LOC changes, despite the fact that 66% of all DC gene pairs in AD exhibit GOC (**Figure 1** in the main text). For example, all of the 85 genes connected to *APOE* and 129 (87%) of the genes connected to *SASH1* show LOC in AD (**Table 2** in the main text). We checked if differential expression (DE) could explain the loss of coordinate expression of these gene pairs, but found that none of the gene pairs showing LOC were differentially expressed (see more details on the relationship between DE and DC above).

Polymorphism in *HTT* is the predominant genetic cause of HD (Roze et al, 2010), and we find that *HTT* gained two co-expression relationships in HD, i.e. to *INSR* and *NPY1R*. This is of interest given recent data showing that injection of neuropeptide Y (NPY) in mice influences progression of HD (Decressac et al, 2010). Furthermore it is well documented that glucose homeostasis is disturbed in HD (Aziz et al, 2010).

A.3 Hub genes in AD and HD comparisons to controls

The top 10 genes that participated in the largest number of DC pairs in AD were *USH1C*, *SLC39A11*, *GSN*, *TIMELESS*, *GPS2*, *RNASE1*, *CHST6*, *PFKP*, *CSRP1* and *PTN*, and each of these genes gained or lost between 144 and 244 co-expression relations in AD compared to

controls. These genes formed three connected groups based on DC relationships among themselves (**Suppl Figure S5A**): *RNASE1* and *GSN* formed a group that mainly exhibited LOC relationships, *PTN* didn't connect with the other top 10 genes, and the rest formed a predominantly GOC group. The disruptions of *USH1C*, *PTN* and *GSN* have been associated with specific forms of neurodegenerative complications (Keats & Savas, 2004; Mi et al, 2007) or amyloidosis (Maury et al, 1990). *TIMELESS* is a key regulator in circadian rhythm (Sangoram et al, 1998), the disturbance of which is common in AD patients (Weldemichael & Grossberg, 2010), whereas *CHST6* helps maintain corneal transparency (Akama et al, 2000). Previous studies have shown that light sensing neurons degenerate in AD (Hinton et al, 1986), which could further disrupt circadian rhythm (Weldemichael & Grossberg, 2010). *PFKP*, a highly expressed phosphofructokinase in the brain (Hannemann et al, 2005), is involved in glycolysis and associated with obesity in GWAS (Scuteri et al, 2007); *SLC39A11* is a zinc transporter (Mocchegiani et al, 2008) and zinc subcellular distribution is a key contributing factor for T2D (Mocchegiani et al, 2008); and finally *GPS2* mediates anti-inflammatory action of key lipid metabolism regulators *LRH-1* and *LXRbeta* (Venteclef et al, 2010). Obesity, T2D and inflammation have been shown to link with each other via highly interconnected molecular networks (Chen et al, 2008; Emilsson et al, 2008).

For HD vs. controls, the genes *PARP11*, *STARD7*, *SNX27*, *EIF2B4*, *AMPD2*, *PAIP1*, *MAN2A2*, *NCOA6*, *UBAP1* and *GPS2* were the top 10 genes with the largest number of DC partners, which ranged from 339 to 550. All these genes participated in GOC relations and were interconnected amongst themselves (**Suppl Figure S5B**). Protein translation is dysregulated in many neurodegenerative diseases including HD (Chang et al, 2006), and we observed several of these top 10 genes as linked to protein synthesis and post-translational protein modifications. For

instance, *EIF2B* regulates protein synthesis responding to multiple stress signals and is associated with AD, HD and Parkinson disease (Chang et al, 2006); *UBAP1* is involved in protein ubiquitination and is linked to a neurodegenerative disease FTLD (Frontotemporal lobe dementia) (Jellinger, 2009); and *PAIP1* interacts with poly(A)-binding protein and cap-binding complex *EIF4A* to regulate translation initiation and protein synthesis (Martineau et al, 2008). Further, *MAN2A2* and *PARP11* are both involved in post-translational protein modifications, and a sorting nexin *SNX27* regulates trafficking of neural potassium channels into different cellular compartments for degradation (Lunn et al, 2007).

When considering only disrupted pairs that are common to both AD and HD, the top 10 genes with the most numbers of DC partners were *RNASE1*, *GSN*, *SLC39A11*, *GPS2*, *CSRP1*, *FAM59B*, *TIMELESS*, *EZR*, *AMPD2* and *SASH1* (**Suppl Figure S5C**). These genes, six of which are also in the top 10 DC gene list for AD (**Suppl Figure S5A,C**), formed three groups with respect to DC relationships among themselves: *RNASE1* and *GSN* formed a group that mainly exhibited LOC relationships, *EZR* didn't connect with the other top 10 genes, and the rest formed a predominantly GOC group. Disease relevance of some of these genes is discussed in the main text.

A.4 GO Biological Processes enriched in DC modules

Besides testing enrichment of AD (and HD) modules for Ingenuity Pathways (**Suppl Tables S3**,**S4**), we also tested enrichment of modules for GO Biological Processes (of size between 50 and 1000 genes to exclude too small or too broadly defined categories), and discuss here some of the significant enrichments we found at the 0.05 level after Bonferroni correction for the number of GO Biological Processes tested. The enrichment of AD modules for developmental processes

including M4 for striated muscle tissue development (P=2.8e-6) and M20 for skeletal system development (P=5.9e-10) is intriguing in light of the recently established connection between sarcopenia and AD (Burns et al, 2010). The enrichment of neurotransmission related genes (eg. M9 for "Transmission of nerve impulse" at P=8.3e-6) is not unexpected as AD is a disease of synaptic failure (Selkoe, 2002), and the link to inflammation (M23 for "Acute inflammatory response" at P=1.7e-7) is also expected as the hallmark neuropathological lesions and tangles are associated with microglia activation in the central nervous system (Krause & Muller, 2010). Two of the HD modules (M5 and M7) in the top 10 DC modules (ordered based on their modularity scores) detected in HD showed enrichment for protein folding (P=3e-10 and P=2.2e-5 respectively), which is in line with the proposed pathological mechanisms of mutant *HTT* gene expressing deficits in protein folding (Wang et al, 2008), and in mitochondrial energy generation (Browne, 2008).

Since GOC pairs dominate LOC pairs in the overall DC network, it was interesting to observe that some of the DC modules with predominantly LOC pairs (at least 75% of all DC pairs) were enriched for biological processes related to homophilic cell adhesion (AD M144 at P=1e-18, and HD M18 at P=2.2e-30) and lipid-related processes (AD M3 at P=1.9e-5, and HD M106 at P=2.7e-5). The accumulated results suggest that exploring changes in the co-ordinate expression patterns of genes between the normal and disease states can capture major pathways and mechanisms involved in the pathophysiology of the diseases in question.

References

Akama TO, Nishida K, Nakayama J, Watanabe H, Ozaki K, Nakamura T, Dota A, Kawasaki S, Inoue Y, Maeda N, Yamamoto S, Fujiwara T, Thonar EJ, Shimomura Y, Kinoshita S, Tanigami A, Fukuda MN (2000) Macular corneal dystrophy type I and type II are caused by distinct mutations in a new sulphotransferase gene. *Nat Genet* **26:** 237-241

Aziz NA, Pijl H, Frolich M, Snel M, Streefland TC, Roelfsema F, Roos RA (2010) Systemic energy homeostasis in Huntington's disease patients. *J Neurol Neurosurg Psychiatry* **81:** 1233-1237

Beecham GW, Martin ER, Li YJ, Slifer MA, Gilbert JR, Haines JL, Pericak-Vance MA (2009) Genome-wide association study implicates a chromosome 12 risk locus for late-onset Alzheimer disease. *Am J Hum Genet* **84:** 35-43

Bertram L, Lange C, Mullin K, Parkinson M, Hsiao M, Hogan MF, Schjeide BM, Hooli B, Divito J, Ionita I, Jiang H, Laird N, Moscarillo T, Ohlsen KL, Elliott K, Wang X, Hu-Lince D, Ryder M, Murphy A, Wagner SL et al (2008) Genome-wide association analysis reveals putative Alzheimer's disease susceptibility loci in addition to APOE. *Am J Hum Genet* **83:** 623-632

Browne SE (2008) Mitochondria and Huntington's disease pathogenesis: insight from genetic and chemical models. *Ann N Y Acad Sci* **1147:** 358-382

Burns JM, Johnson DK, Watts A, Swerdlow RH, Brooks WM (2010) Reduced lean mass in early Alzheimer disease and its association with brain atrophy. *Arch Neurol* **67:** 428-433

Carrasquillo MM, Zou F, Pankratz VS, Wilcox SL, Ma L, Walker LP, Younkin SG, Younkin CS, Younkin LH, Bisceglio GD, Ertekin-Taner N, Crook JE, Dickson DW, Petersen RC, Graff-Radford NR (2009) Genetic variation in PCDH11X is associated with susceptibility to late-onset Alzheimer's disease. *Nat Genet* **41:** 192-198

Chang RC, Yu MS, Lai CS (2006) Significance of molecular signaling for protein translation control in neurodegenerative diseases. *Neurosignals* **15:** 249-258

Chen Y, Zhu J, Lum PY, Yang X, Pinto S, MacNeil DJ, Zhang C, Lamb J, Edwards S, Sieberts SK, Leonardson A, Castellini LW, Wang S, Champy MF, Zhang B, Emilsson V, Doss S, Ghazalpour A, Horvath S, Drake TA et al (2008) Variations in DNA elucidate molecular networks that cause disease. *Nature* **452:** 429-435

Decressac M, Wright B, Tyers P, Gaillard A, Barker RA (2010) Neuropeptide Y modifies the disease course in the R6/2 transgenic model of Huntington's disease. *Exp Neurol* **226**

Emilsson V, Thorleifsson G, Zhang B, Leonardson AS, Zink F, Zhu J, Carlson S, Helgason A, Walters GB, Gunnarsdottir S, Mouy M, Steinthorsdottir V, Eiriksdottir GH, Bjornsdottir G, Reynisdottir I, Gudbjartsson D, Helgadottir A, Jonasdottir A, Styrkarsdottir U, Gretarsdottir S et al (2008) Genetics of gene expression and its effect on disease. *Nature* **452:** 423-428

Hannemann A, Jandrig B, Gaunitz F, Eschrich K, Bigl M (2005) Characterization of the human P-type 6-phosphofructo-1-kinase gene promoter in neural cell lines. *Gene* **345:** 237-247

Harold D, Abraham R, Hollingworth P, Sims R, Gerrish A, Hamshere ML, Pahwa JS, Moskvina V, Dowzell K, Williams A, Jones N, Thomas C, Stretton A, Morgan AR, Lovestone S, Powell J, Proitsi P, Lupton MK, Brayne C, Rubinsztein DC et al (2009) Genome-wide association study

identifies variants at CLU and PICALM associated with Alzheimer's disease. *Nat Genet* **41:** 1088-1093

Heinzen EL, Need AC, Hayden KM, Chiba-Falek O, Roses AD, Strittmatter WJ, Burke JR, Hulette CM, Welsh-Bohmer KA, Goldstein DB (2009) Genome-wide scan of copy number variation in late-onset Alzheimer's disease. *J Alzheimers Dis* **19:** 69-77

Hinton DR, Sadun AA, Blanks JC, Miller CA (1986) Optic-nerve degeneration in Alzheimer's disease. *N Engl J Med* **315:** 485-487

Jellinger KA (2009) Recent advances in our understanding of neurodegeneration. *J Neural Transm* **116:** 1111-1162

Keats BJ, Savas S (2004) Genetic heterogeneity in Usher syndrome. *Am J Med Genet A* **130A:** 13-16

Kramer PL, Xu H, Woltjer RL, Westaway SK, Clark D, Erten-Lyons D, Kaye JA, Welsh-Bohmer KA, Troncoso JC, Markesbery WR, Petersen RC, Turner RS, Kukull WA, Bennett DA, Galasko D, Morris JC, Ott J (2010) Alzheimer disease pathology in cognitively healthy elderly: A genome-wide study. *Neurobiol Aging*

Krause DL, Muller N (2010) Neuroinflammation, microglia and implications for anti-inflammatory treatment in Alzheimer's disease. *Int J Alzheimers Dis* **2010**

Lopez OL, Lopez-Pousa S, Kamboh MI, Adroer R, Oliva R, Lozano-Gallego M, Becker JT, DeKosky ST (1998) Apolipoprotein E polymorphism in Alzheimer's disease: a comparative study of two research populations from Spain and the United States. *Eur Neurol* **39:** 229-233

Lunn ML, Nassirpour R, Arrabit C, Tan J, McLeod I, Arias CM, Sawchenko PE, Yates JR, 3rd, Slesinger PA (2007) A unique sorting nexin regulates trafficking of potassium channels via a PDZ domain interaction. *Nat Neurosci* **10:** 1249-1259

Martineau Y, Derry MC, Wang X, Yanagiya A, Berlanga JJ, Shyu AB, Imataka H, Gehring K, Sonenberg N (2008) Poly(A)-binding protein-interacting protein 1 binds to eukaryotic translation initiation factor 3 to stimulate translation. *Mol Cell Biol* **28:** 6658-6667

Maury CP, Kere J, Tolvanen R, de la Chapelle A (1990) Finnish hereditary amyloidosis is caused by a single nucleotide substitution in the gelsolin gene. *FEBS Lett* **276:** 75-77

Mi R, Chen W, Hoke A (2007) Pleiotrophin is a neurotrophic factor for spinal motor neurons. *Proc Natl Acad Sci U S A* **104:** 4664-4669

Mocchegiani E, Giacconi R, Malavolta M (2008) Zinc signalling and subcellular distribution: emerging targets in type 2 diabetes. *Trends Mol Med* **14:** 419-428

Reiman EM, Webster JA, Myers AJ, Hardy J, Dunckley T, Zismann VL, Joshipura KD, Pearson JV, Hu-Lince D, Huentelman MJ, Craig DW, Coon KD, Liang WS, Herbert RH, Beach T, Rohrer KC, Zhao AS, Leung D, Bryden L, Marlowe L et al (2007) GAB2 alleles modify Alzheimer's risk in APOE epsilon4 carriers. *Neuron* **54:** 713-720

Roze E, Bonnet C, Betuing S, Caboche J (2010) Huntington's disease. *Adv Exp Med Biol* **685:** 45-63

Sangoram AM, Saez L, Antoch MP, Gekakis N, Staknis D, Whiteley A, Fruechte EM, Vitaterna MH, Shimomura K, King DP, Young MW, Weitz CJ, Takahashi JS (1998) Mammalian circadian autoregulatory loop: a timeless ortholog and mPer1 interact and negatively regulate CLOCK-BMAL1-induced transcription. *Neuron* **21:** 1101-1113

Scuteri A, Sanna S, Chen WM, Uda M, Albai G, Strait J, Najjar S, Nagaraja R, Orru M, Usala G, Dei M, Lai S, Maschio A, Busonero F, Mulas A, Ehret GB, Fink AA, Weder AB, Cooper RS, Galan P et al (2007) Genome-wide association scan shows genetic variants in the FTO gene are associated with obesity-related traits. *PLoS Genet* **3:** e115

Selkoe DJ (2002) Alzheimer's disease is a synaptic failure. *Science* **298:** 789-791

Tsai MS, Tangalos EG, Petersen RC, Smith GE, Schaid DJ, Kokmen E, Ivnik RJ, Thibodeau SN (1994) Apolipoprotein E: risk factor for Alzheimer disease. *Am J Hum Genet* **54:** 643-649

Venteclef N, Jakobsson T, Ehrlund A, Damdimopoulos A, Mikkonen L, Ellis E, Nilsson LM, Parini P, Janne OA, Gustafsson JA, Steffensen KR, Treuter E (2010) GPS2-dependent corepressor/SUMO pathways govern anti-inflammatory actions of LRH-1 and LXRbeta in the hepatic acute phase response. *Genes Dev* **24:** 381-395

Wang J, Wang CE, Orr A, Tydlacka S, Li SH, Li XJ (2008) Impaired ubiquitin-proteasome system activity in the synapses of Huntington's disease mice. *J Cell Biol* **180:** 1177-1189

Weldemichael DA, Grossberg GT (2010) Circadian rhythm disturbances in patients with Alzheimer's disease: a review. *Int J Alzheimers Dis* **2010**

B. Supplementary Tables

Suppl Table S1. Characteristics of the disease and control populations used in this study. PMI stands for Post-Mortem Interval in hours. Age is in years. Only individuals whose DLPFC (dorsolateral prefrontal cortex) expression data passed quality control are included here.

Group	Number of subjects	Males / Females	Mean Age (SD)	Mean PMI (SD)
All	624	341 / 283	70.1 (15.3)	17.5 (8.2)
AD patients	310	135 / 175	80.6 (9.0)	13.7 (7.4)
HD patients	157	83 / 74	55.9 (14.7)	20.0 (7.9)
Controls	157	123 / 34	63.5 (9.9)	22.4 (5.8)

Suppl Table S2. Results from the replication testing of known GWAS risk loci for AD in the HBTRC samples. The number of AD cases was 375 and the number of controls 156. We used a logistic regression model, adjusting for age and gender. The results that we consider significant at a significance level of 0.1 are marked in bold. Published GWAS results for AD can be found at www.genome.gov/gwastudies.

SNP	Effect allele	OR	P-value	Implicated gene
APOE ε4	ε4	3.73	**4.1e-13**	APOE
rs2373115	A	1.62	**0.004**	GAB2
rs4298437	T	0.88	0.517	RELN
rs11782819	T	1.10	0.607	MSRA
rs690705	A	1.01	0.963	RFC3
rs7539409	G	1.33	0.298	TTLL7
rs4509693	C	0.79	0.275	PAX2
rs9390537	C	1.50	**0.084**	SASH1
rs11136000	T	0.80	0.240	CLU
rs5984894 (proxy of rs2573905)	A	0.99	0.964	PCDH11X
rs11610206	C	2.01	**0.044**	FAM113B
rs2061333	C	0.80	0.361	ZNF224
rs11122322 (proxy of rs12044355)	G	1.12	0.547	DISC1
rs3826656	G	1.06	0.780	CD33
rs727153	T	0.96	0.808	Intergenic
rs3851179	T	1.16	0.428	PICALM
rs3752246	G	NA	NA	ABCA7
rs7561528	A	1.25	0.258	BIN1
rs2588963 (proxy of rs2588969)	A	1.07	0.688	ARID5B
rs6701713	A	1.21	0.408	CR1
rs4938933	C	0.88	0.484	MS4A4A

Suppl Table S3. Enrichment of the DC modules in AD vs. Controls comparison for curated pathways in Ingenuity Pathways (www.ingenuity.com). The enrichments were significant at the 0.05 level after Bonferroni correction for the number (182) of Ingenuity Pathways tested, and the best enrichment is shown for any module with multiple significant enrichments. The hypergeometric enrichment P-values are computed using a background of 19,198 genes. The symbol **N** stands for "Number of genes".

**Module**	**Ingenuity Pathway**	**Enrich-ment P-value**	**N genes in module**	**N genes in overlap**	**N genes in pathway**
M1	Histidine Metabolism	1.5e-05	69	4	42
M9	cAMP-mediated Signaling	2.5e-05	73	6	152
M17	Hepatic Cholestasis	0.00022	81	5	129
M20	Hepatic Fibrosis Hepatic Stellate Cell Activation	3.9e-05	58	5	126
M32	Arginine and Proline Metabolism	2.3e-05	45	4	71
M40	Antigen Presentation Pathway	0.00016	64	3	32
M45	Amyloid Processing	1.1e-05	53	4	50
M61	Valine Leucine and Isoleucine Biosynthesis	0.00022	36	2	12
M75	Role of Pattern Recognition Receptors in Recognition of Bacteria and Viruses	3.8e-07	37	5	78
M77	Circadian Rhythm Signaling	1.7e-07	37	4	26
M81	Sulfur Metabolism	0.00021	28	2	15
M89	NRF2-mediated Oxidative Stress Response	3.7e-05	22	4	168
M137	Hepatic Fibrosis Hepatic Stellate Cell Activation	2.3e-05	9	3	126
M146	Ubiquinone Biosynthesis	0.00018	8	2	50

Suppl Table S4. Enrichment of the DC modules in HD vs. Controls comparison for Ingenuity pathways, reported using the same criteria as in **Suppl Table S3**.

Module	Ingenuity Pathway	Enrich-ment P-value	N genes in module	N genes in overlap	N genes in pathway
M15	Starch and Sucrose Metabolism	0.00021	74	4	76
M19	Folate Biosynthesis	1.2e-05	69	3	13
M22	Nitrogen Metabolism	1.4e-05	71	4	40
M24	Lysine Degradation	4.5e-05	59	4	64
M27	Propanoate Metabolism	7.4e-05	74	4	58
M40	Neuregulin Signaling	0.00027	73	4	82
M41	Fc Epsilon RI Signaling	6.8e-05	88	5	93
M42	Calcium Signaling	6.1e-05	79	6	165
M75	LXR RXR Activation	9.3e-05	68	4	67
M108	Complement System	0.00022	69	3	33
M120	p53 Signaling	0.00011	55	4	87
M125	Glutamate Receptor Signaling	0.00024	42	3	55
M146	Fatty Acid Metabolism	1.7e-05	31	4	97
M151	Calcium Signaling	0.00022	35	4	165
M192	Circadian Rhythm Signaling	0.00012	12	2	26
M219	p38 MAPK Signaling	5.7e-06	8	3	91

Suppl Table S5. Enrichment of DC modules identified in AD vs. controls comparison for genes correlated to AD Braak stage severity. The enrichments were significant at the 0.05 level after Bonferroni correction for the number of tested DC modules. The expression profiles of 780 of the 39,909 gene transcripts in the HBTRC samples were correlated to Braak stage at a Pearson correlation $P < 0.01$ (after Bonferroni correction for the number of tested probes; the probes' expression data used here was adjusted as described in the main text but without including age as a covariate to retain the relationship between age, Braak stage and gene expression). The symbol **N** stands for "Number of".

DC module	Enrichment P-value (P)	N Braak-correlated genes in module	N genes in module
AD/M6	8.1e-05	9	92
AD/M39	1.3e-4	7	58
AD/M45	1.1e-06	10	70
AD/M50	2.7e-05	8	62

C. Supplementary Figures

Suppl Figure S1. Differential Expression (DE) and Differential Co-expression (DC) analysis results in complementary sets of genes. The results are shown for the (**A**) AD vs. controls and (**B**) HD vs. controls comparisons as 3D histograms. The distribution of the number of detected GOC or LOC pairs (z axis) is plotted against the logarithm of the t-statistic based DE P-values of the genes in the pairs (x, y axes, with more differentially expressed gene in a pair shown in the y axis labeled $\log_{10}$(DE Pvalue j)).

A

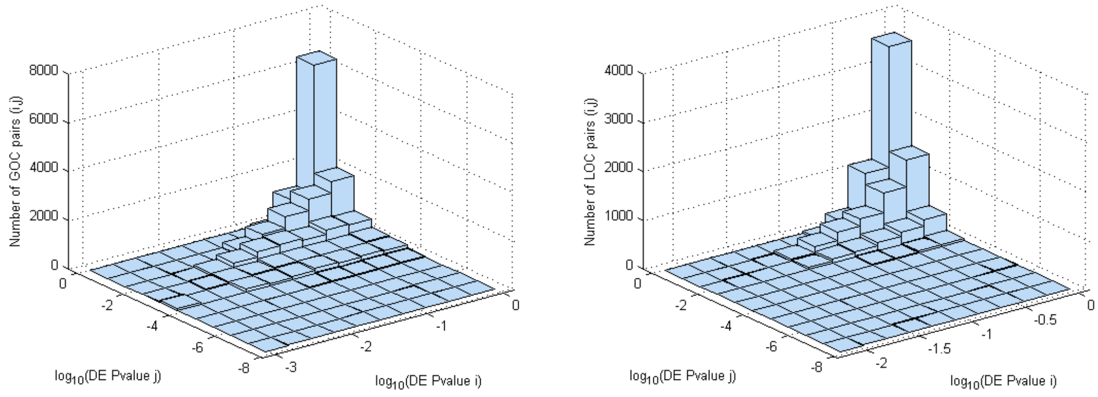

B

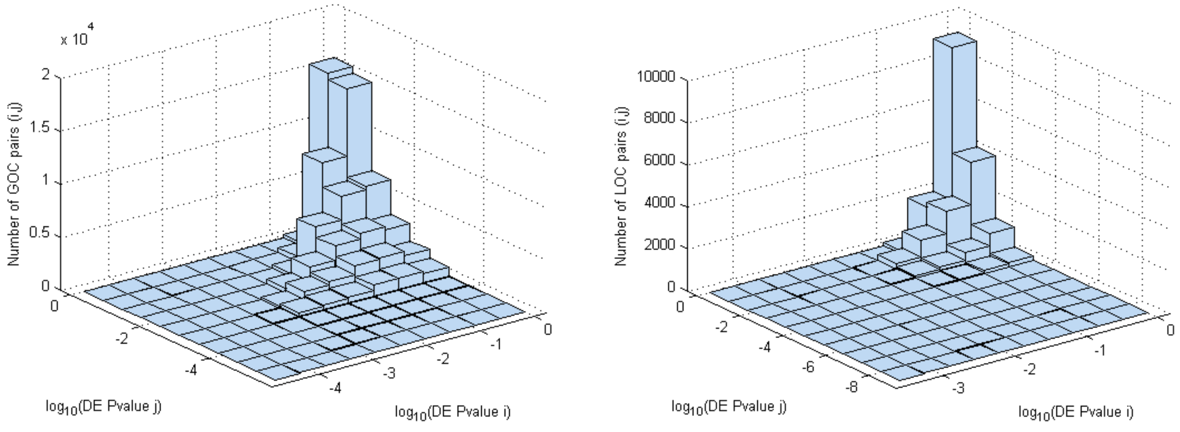

Suppl Figure S2. Differential Expression (DE) and Differential Co-expression (DC) analysis results in complementary sets of genes in AD vs. controls comparison. (**A**) Each reporter's t-statistic (x-axis) is plotted against the number of GOC pairs in which the reporter participates (GOC neighbors) minus the number of LOC pairs in which the reporter participates (LOC neighbors) in the y-axis. The lines drawn correspond to t-statistic values of -1.96 and1.96 (translating to a DE P-value of ~0.05), and differences in the GOC and LOC neighbors of -5 and 5 (chosen arbitrarily). (**B**) For each detected GOC and LOC pair, the Q-statistic of the pair (x-axis) is plotted against the minimum of the absolute t-statistic values of each reporter in the pair (y-axis). The line shown corresponds to the minimum absolute t-statistic value of 1.96 (translating to a DE P-value of ~0.05).

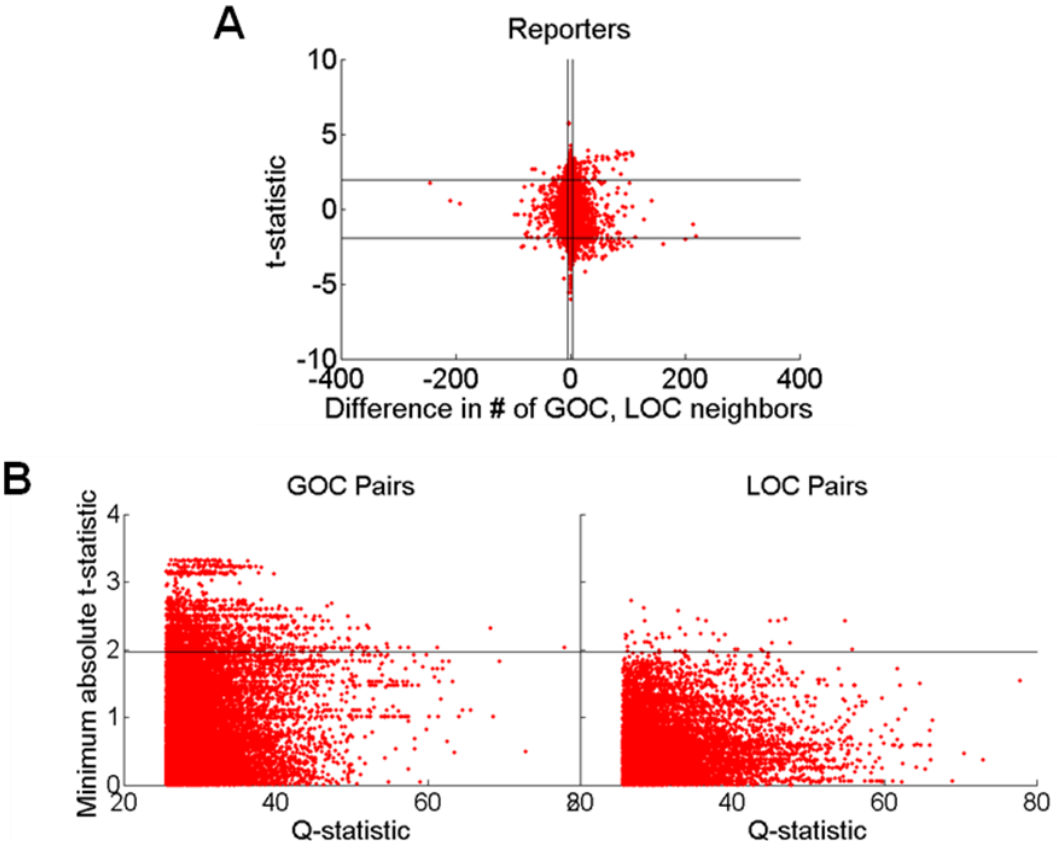

Suppl Figure S3. Differential Expression (DE) and Differential Co-expression (DC) analysis results in complementary sets of genes in HD vs. controls comparison. The axes and conventions used in these figures are the same as in **Suppl Figure S2**. As panel **B** shows, at a lenient (uncorrected) DE P-value cutoff of 0.05, we found that 10.29% of all 84,541 GOC pairs were between two DE reporters, whereas only 1.00% of all 21,593 LOC pairs were between two DE reporters (these values are 44.66% and 25.49% respectively if we require only one reporter in the GOC or LOC pair to be DE).

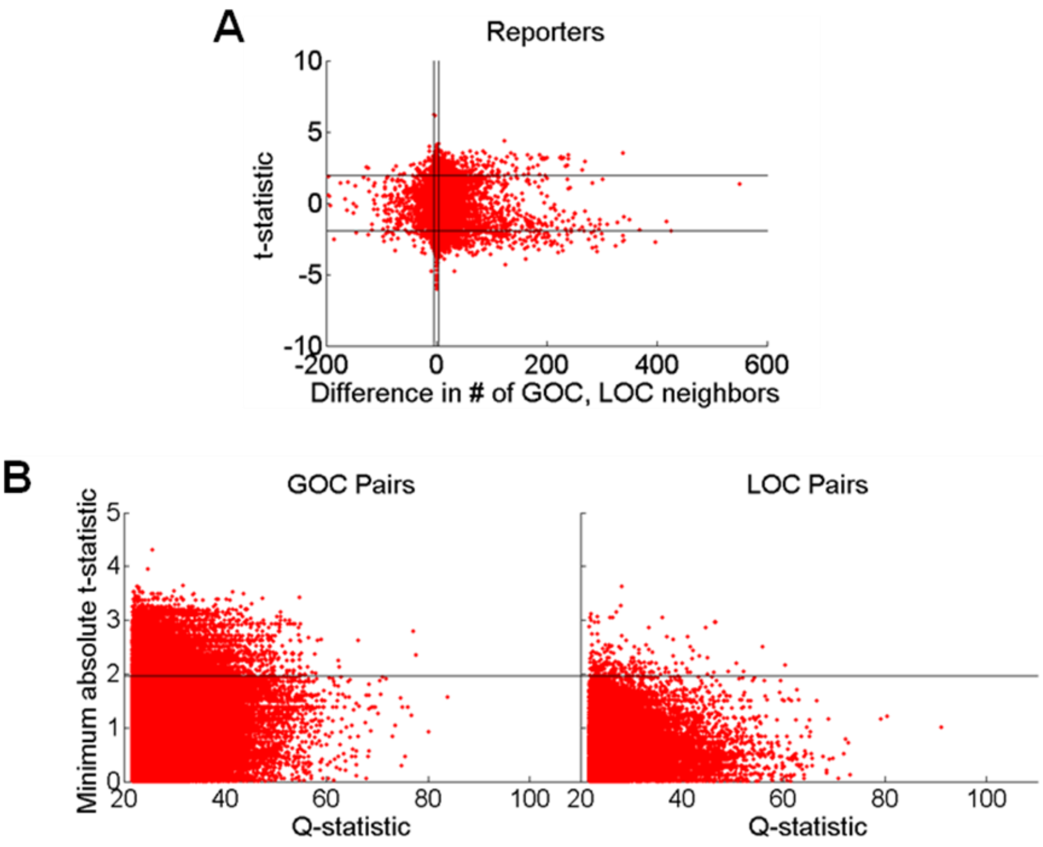

Suppl Figure S4. Spot with axis label (i/10,j/10) in a heatmap shows the plotted value of a reporter pair (i,j) i.e., the ith,jth reporters in a DC network or module. The GOC and LOC pairs are indicated respectively as dark and gray spots in the DC network and DC module. (**A**) The AD DC network is clustered and the resulting reordering of reporters is used to plot these heatmaps. The clustering structure is more evident from the Q-statistics plot. **(B)** We highlight the DC module M9 obtained from (**A**), shown as a red dot in (**A**) near spot 5000,5000, to show the DC pairs in this module and the correlation coefficient changes between all reporter pairs in this module. The upper half of the absolute ρ (Spearman correlation coefficient) heatmap is based on correlation of reporter pairs in the AD disease samples and its lower half is based on correlation in the controls. Darker spots in the absolute ρ or Q-statistics heatmaps reflect larger values – for instance, the shading in the absolute ρ heatmap shows many reporter pairs in this module are LOC.

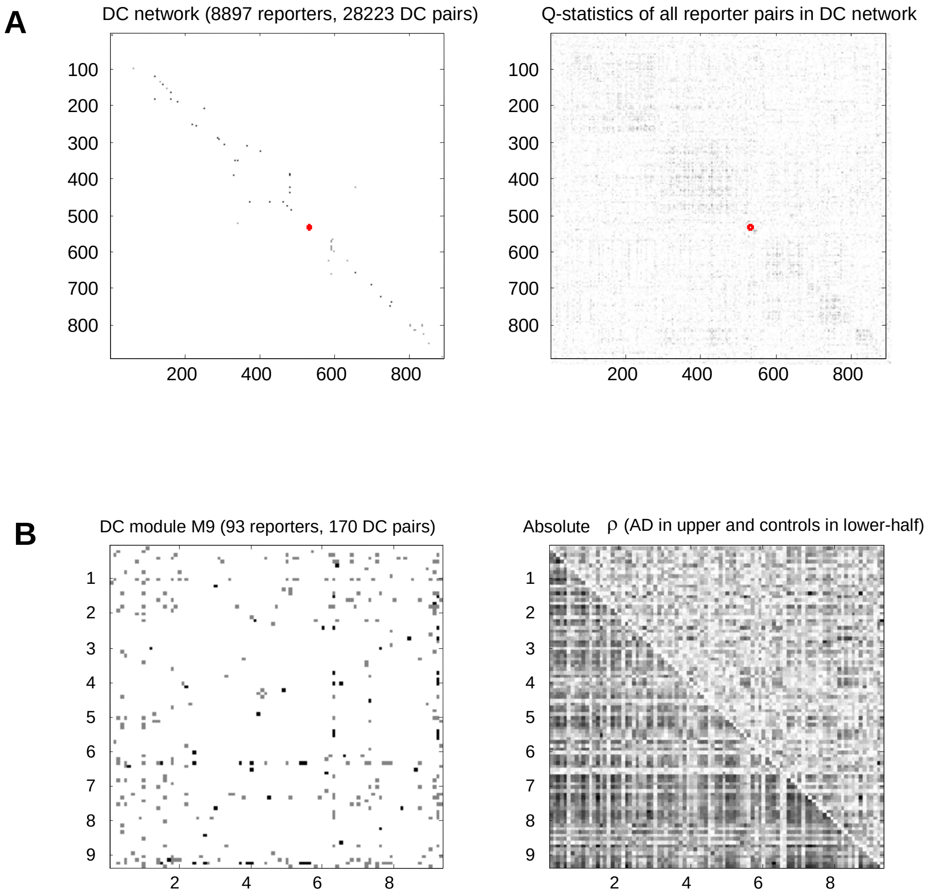

Suppl Figure S5. Connectivity between the ten genes with the most number of neighbors in the DC network from: (**A**) AD vs. Controls, (**B**) HD vs. Controls, and (**C**) common to both comparisons. Red edges indicate GOC pairs among the nodes, and red nodes indicate genes with at least 80% of their DC pairs being GOC. Green color is indicative of LOC relations.

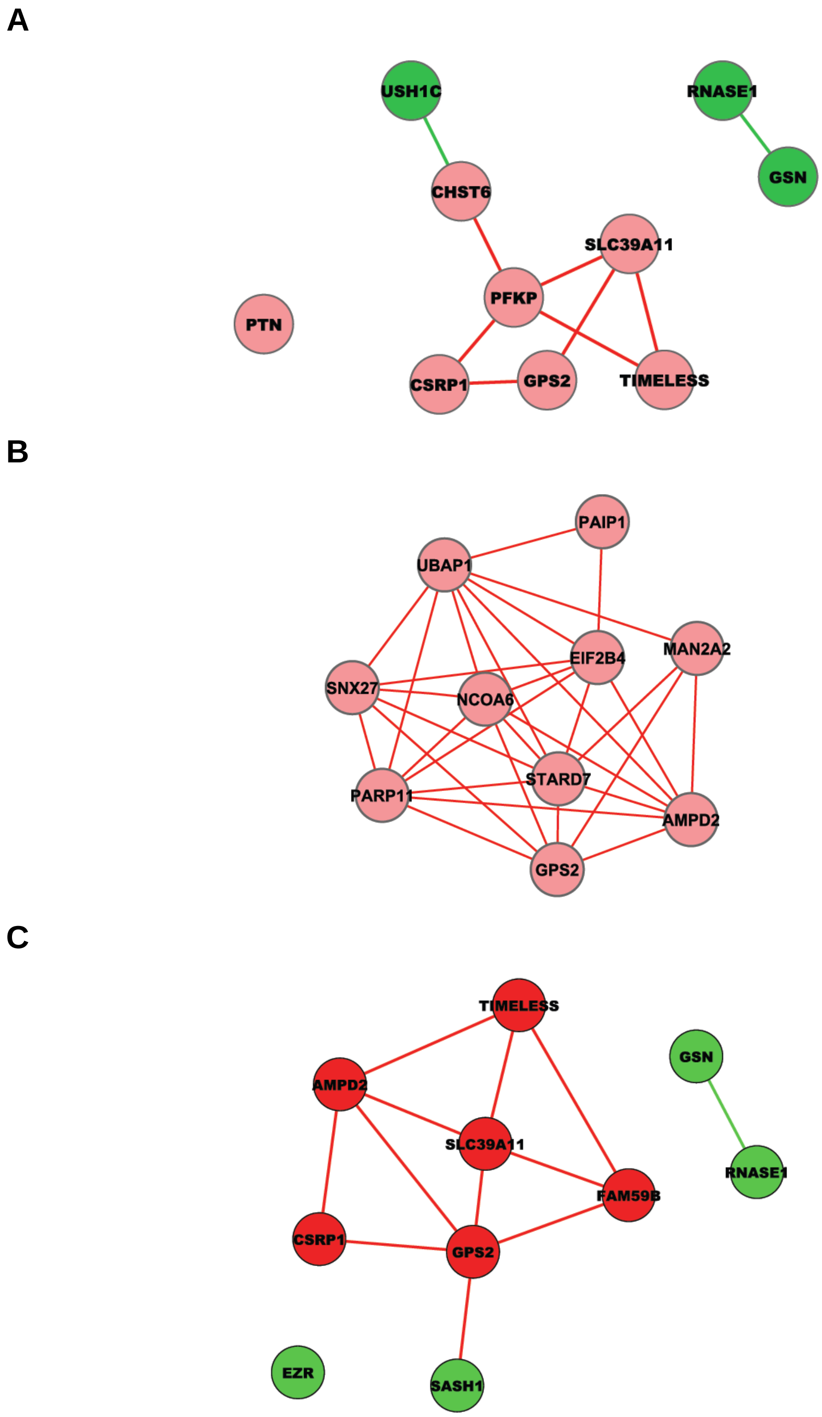

Suppl Figure S6. (A) Among the DC pairs we detected in the AD vs. controls HBTRC samples at various Q cutoffs (denoted AD DC pairs, with the black line marking the chosen 1% FDR cutoff) and that had representation in the independent AD and aging datasets, a significantly higher fraction was replicated in the independent data (in terms of both GOC/LOC direction and magnitude of Q as described in **Figure 2C** in the main text, but using additional Q cutoffs in the independent data besides 3.84 to call replication) when compared to 1000 matched sets of random pairs of the same size and network connectivity (obtained as in the main text). Each dark curve corresponds to the replication fraction of the detected DC pairs and the 10 lighly shaded curves for each dark curve shows the same fraction with 10 matched sets of random gene pairs. **(B)** Similar trends were seen after removal of age-associated DC pairs from the HBTRC DC pairs (denoted AD-Aging). **(C & D)** The replication fractions for all 1000 matched sets of random DC pairs along with a line indicating the same fraction for the detected set of DC pairs at 1% FDR are shown for AD and AD-Aging DC pairs in **(C)** and **(D)** respectively for the Q > 3.84 cutoff in the independent data**,** as three histograms corresponding to DC, GOC and LOC pairs in that order.

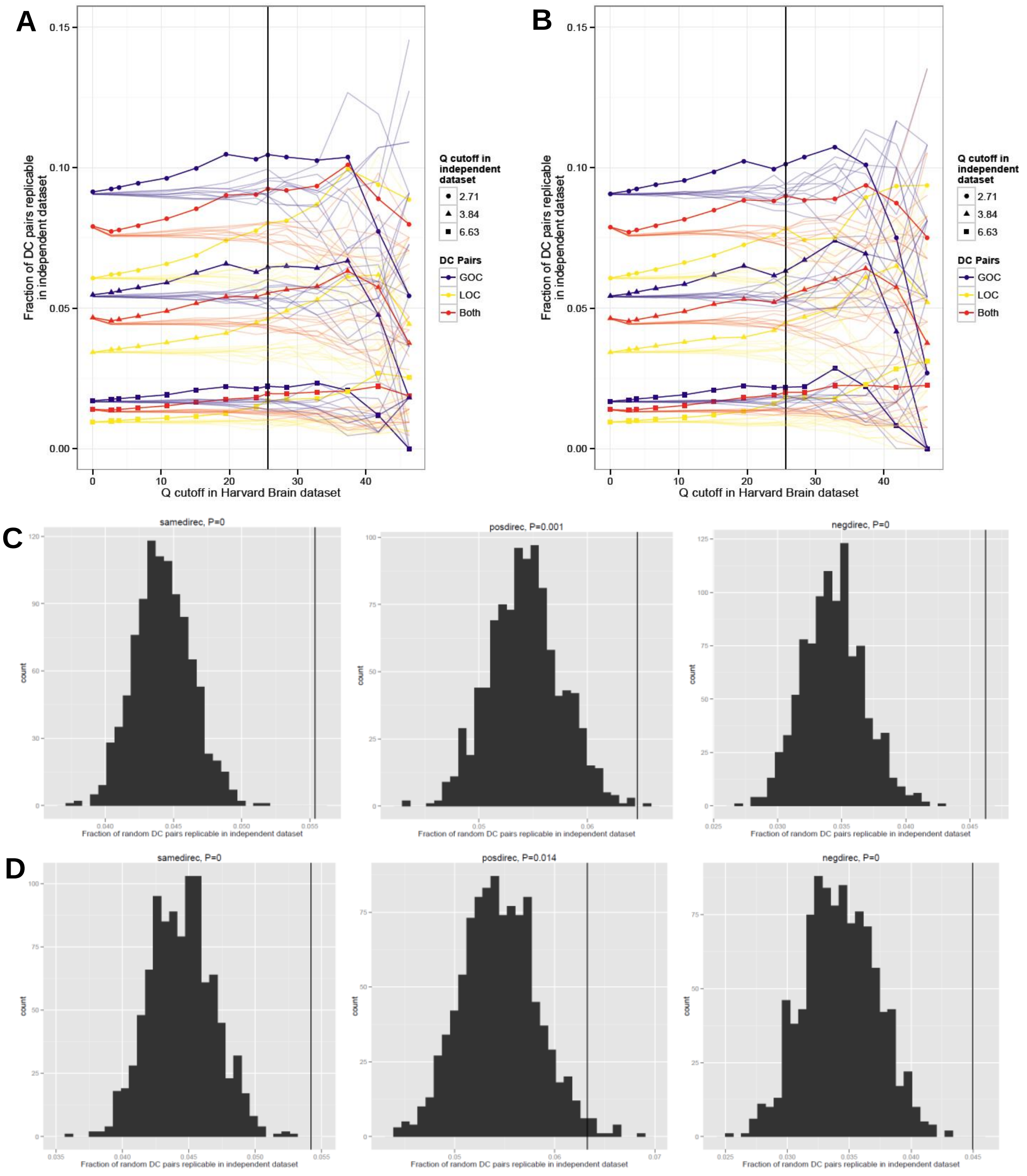

Suppl Figure S7. Comparison of the parametric meta-analysis method (based on Q statistic) to the non-parametric bootstrap method, for making DC calls from a random subset of all gene pairs in the AD vs. controls comparison. The two methods yield similar results, with the P-value P_Q based on the analytical null distribution of the Q-statistic (which is Chi-squared with one degree of freedom) somewhat more conservative than the P-value based on the bootstrap method (P_bootstrap). This trend holds for most P-values, except for very small P-values that are not resolved by the bootstrap method due to the 1/1000 limit imposed by the 1000 bootstrap resamplings we do. Since it is computationally intensive to perform several bootstrap resamplings per gene pair, the plot shows results only for a random subset of all gene pairs obtained by pairing up a random set of 100 genes (probes) with all other probes profiled on the microarray.
Note that the bootstrap method yields two P-values for any given gene pair based on which group (disease or controls) is resampled, and P_bootstrap is taken as the average of these two P-values. That is, comparing a gene pair's correlation coefficient in the control samples to a bootstrap distribution of its correlation coefficient in 1000 resamplings of the disease samples yields a disease-bootstrap P-value (that is twice the one-sided empirical P-value), and repeating this procedure *vice versa* yields a controls-bootstrap P-value for the same gene pair.

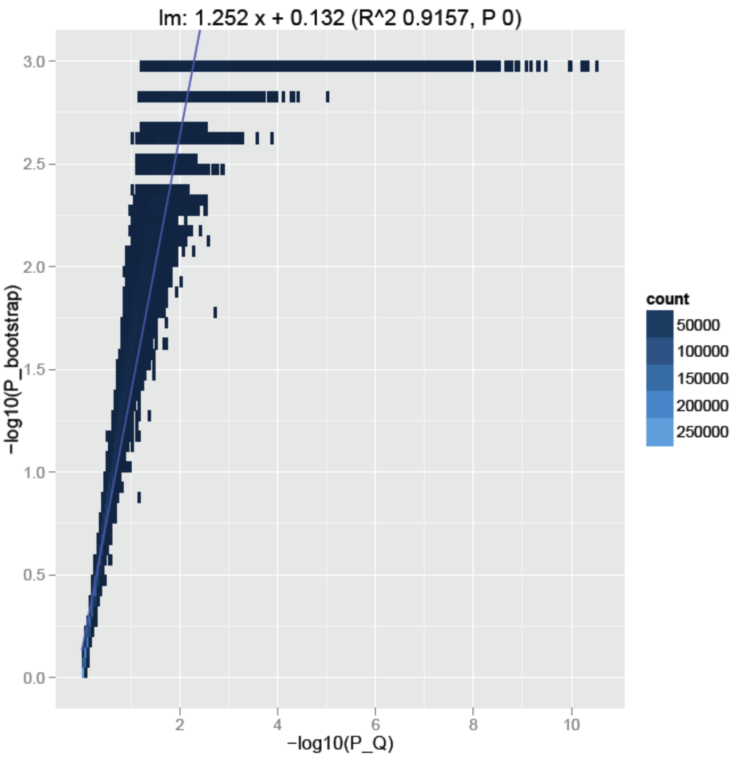

Suppl Figure S8. Confocal images of cells stained with lineage-specific antibodies and antibodies for Dnmt1 and Dnmt3a. Green: cell markers' stains, and red: Dnmt1 and Dnmt3a stains. *Dnmt1* (A) and *Dnmt3a* (B) were efficiently knocked out in oligodendrocyte cells. The white arrows indicate absence of the enzymes Dnmt1 and Dnmt3a only in the nucleus of oligodendrocytes (Olig2+), but not in the nucleus of astrocytes (GFAP+) or neurons (NeuN+). Scale bar: 5μm.

A

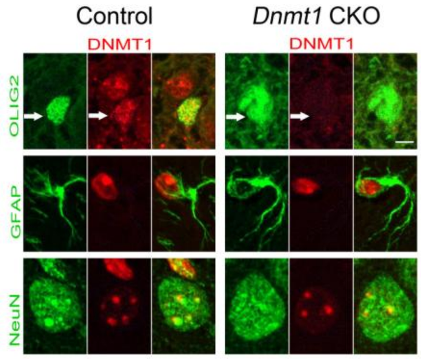

B

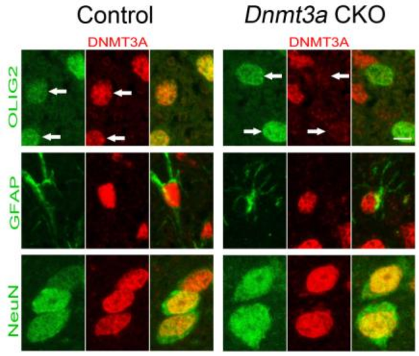

D. Supplementary Datasets

Please note that the datasets described here are provided in separate supporting files.

Suppl Dataset D1. The network of DC (GOC+LOC) gene pairs detected in the AD vs. Controls (a), or HD vs. Controls (b) comparison using the HBTRC data.

Suppl Dataset D2. The gene modules resulting from clustering the DC network for AD (a) or HD (b).

Suppl Dataset D3. The 242-gene subnetwork resulting from aligning the DC network common to AD and HD with the physical network of protein-protein and protein-DNA interactions.

Suppl Dataset D4. *Dnmt1* brain-specific knockout signature genes defined by Cuffdiff at q-value < 0.1.

Suppl Dataset D5. *Dnmt3a* brain-specific knockout signature genes defined by Cuffdiff at q-value < 0.1.

Suppl Dataset D6. Transcript and gene annotations of the microarray platform used in this study, which supplement the information available in GEO under platform accession GPL4372. All analyses in our study is based on the conservative annotations in the gene symbol column in this file, with one exception – the replication analysis uses the first annotation in the alternate symbols column to maximize the overlapping gene probes between our HBTRC expression dataset and the two independent brain expression datasets (viz., the aging and AD datasets used in testing replication).
